# Choose your meals carefully if you need to coexist with a toxic invader

**DOI:** 10.1038/s41598-020-78979-8

**Published:** 2020-12-14

**Authors:** Lachlan Pettit, Georgia Ward-Fear, Richard Shine

**Affiliations:** 1 School of Life and Environmental Sciences, University of Sydney, Sydney, NSW 2006 Australia; 2 Department of Biological Sciences, Macquarie University, Sydney, NSW 2019 Australia

**Keywords:** Behavioural ecology, Conservation biology, Invasive species

## Abstract

Vulnerable native species may survive the impact of a lethally toxic invader by changes in behaviour, physiology and/or morphology. The roles of such mechanisms can be clarified by standardised testing. We recorded behavioural responses of monitor lizards (*Varanus panoptes* and *V. varius*) to legs of poisonous cane toads (*Rhinella marina*) and non-toxic control meals (chicken necks or chicken eggs and sardines) along 1300 and 2500 km transects, encompassing the toad’s 85-year invasion trajectory across Australia as well as yet-to-be-invaded sites to the west and south of the currently colonised area. Patterns were identical in the two varanid species. Of monitors that consumed at least one prey type, 96% took control baits whereas toad legs were eaten by 60% of lizards in toad-free sites but 0% from toad-invaded sites. Our survey confirms that the ability to recognise and reject toads as prey enables monitor lizards to coexist with cane toads. As toxic invaders continue to impact ecosystems globally, it is vital to understand the mechanisms that allow some taxa to persist over long time-scales.

## Introduction

Invasive species are a major threat to native taxa in many parts of the world, and have driven many endemic taxa to extinction^1,2^. However, invaders often depress the abundance of a native taxon without completely extirpating it; and in some cases, the affected taxon eventually recovers to coexist with the invader^3,4^. What mechanisms confer this increased tolerance of the invader’s presence? The answer may involve behavioural avoidance of the novel threat, whether it be a predator, a competitor, or a toxic prey item or selective consumption of less toxic body parts of the prey^5,6^. Alternatively, a vulnerable native species may evolve shifts in physiology (e.g., toxin tolerance) or morphology (e.g., relative head size, and thus maximum ingestible prey size) that render an individual less vulnerable to an encounter with the invader^7,8^.

All three of these pathways (changes in behaviour, physiology and morphology) have been reported in the case of native species impacted by the spread of cane toads (*Rhinella marin*a) through Australia. For example, the arrival of toads resulted in blacksnakes (*Pseudechis porphyriacus*) becoming less likely to try to eat a toad, less affected by the anuran’s toxin, and less capable of swallowing a toad large enough to be fatal^7,9^. The situation is less clear in large monitor lizards (*Varanus* spp.) that are fatally poisoned by consuming toads^10–12^. Lethal toxic ingestion of toads causes rapid declines in varanid abundance (lizards trained to avoid toads have higher survival^13^); and two studies in eastern Australia have reported that monitors in toad-infested areas (but not toad-free areas) refuse to consume toad flesh^14,15^. Hence, it seems likely that monitors survive toad invasion by developing taste aversion (the association of cues with symptoms arising from spoiled or toxic substances^16^) rather than by shifts in morphology (monitors can tear prey items apart, so head size does not constrain ingestible prey size^17^) or physiology (toad-sympatric monitors lack genetic changes that confer toxin resistance^18^).

However, evidence for a broadscale shift towards taste aversion by monitors in toad-colonised areas is weak. Spatial sampling to support this inference rests on comparisons between lizards from localised sites with versus without toads^14,15^. Such comparisons may fail to capture general patterns over broad spatial scales (and thus changes over invasion). To evaluate the generality of taste aversion as a mechanism allowing persistence of monitors following the invasion of cane toads, we conducted an extensive series of taste aversion trials across northern and eastern Australia. We recorded behavioural responses of monitor lizards (*Varanus panoptes* and *V. varius*) to legs of toxic cane toads and non-toxic control meals along 1300 and 2500 km transects, encompassing the toad’s 85-year invasion trajectory across Australia as well as yet-to-be-invaded sites to the west and south of the currently colonised area. We hypothesised that lizards in areas long colonised by toads would avoid eating toxic prey items.

## Materials and methods

**Figure 1:**
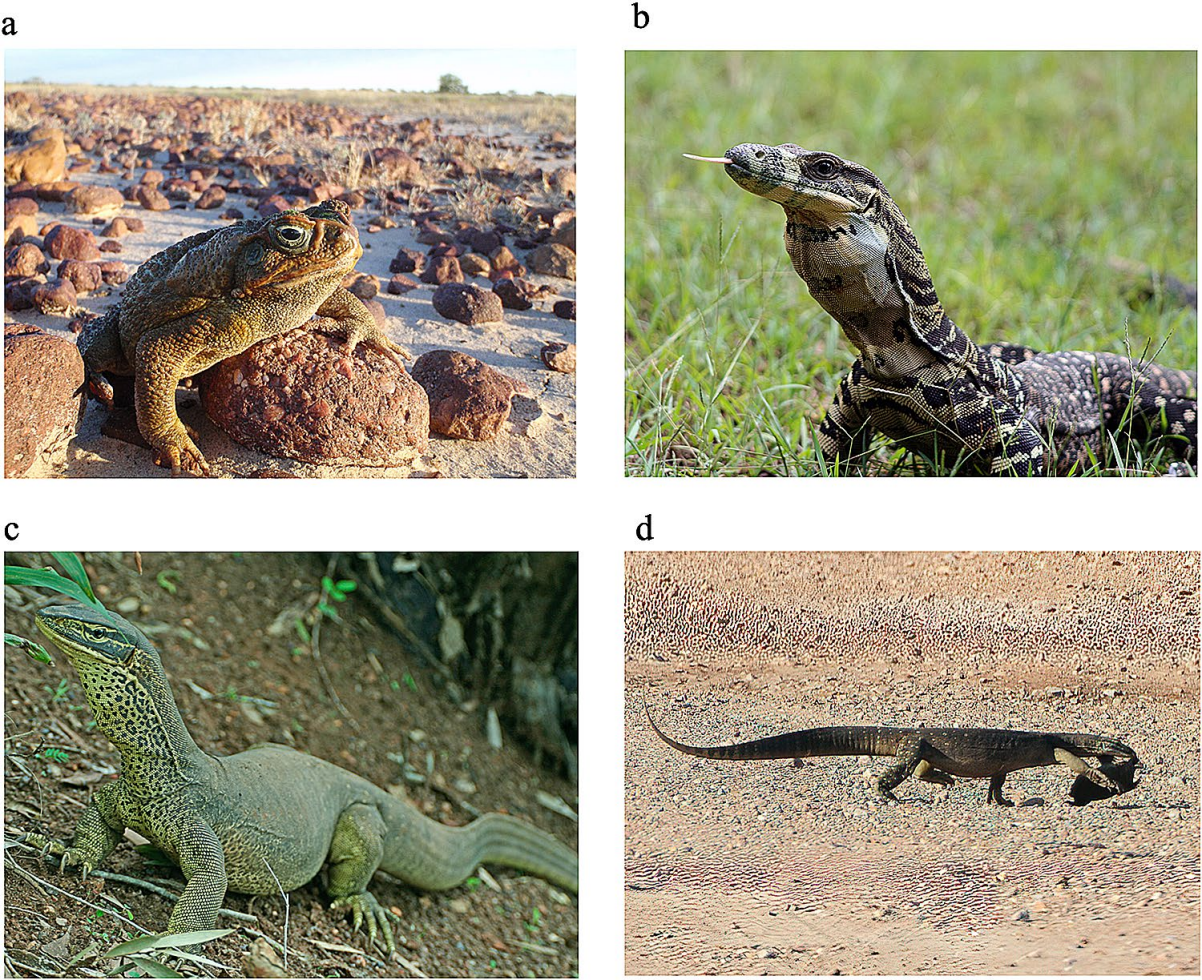
Photographs of **(a)** a cane toad, *Rhinella marina*; **(b)** a lace monitor, *Varanus varius*; **(c)** a yellow-spotted monitor, *Varanus panoptes*; and **(d)** a free-ranging *V. panoptes* carrying a road-killed toad near Yeppoon, Queensland. Images provided by the authors **(a,d)**, Sylvain Dubey **(b)**, and Ruchira Somaweera **(c)**.

Released along the east coast of Australia in 1935 as a biological control, cane toads (see Fig. 1a) have since expanded to occupy over 1.2 million km^2^^19^. The main impact of toads falls on large frog-eating predators that lack resistance to the toads’ toxins, and thus die when they consume a toad^20^. As toads spread south they encountered large varanid lizards (lace monitors, *Varanus varius*; see Fig. 1b) along the east coast of Australia^21^. Populations of lace monitors are negatively impacted at the invasion front^22^, but the species remains common in many long-colonised sites^23^. The toad expansion to the west brought them into contact with another large monitor species (yellow-spotted monitors, *Varanus panoptes*; see Fig. 1c) that experienced massive (> 90%) population declines due to ingestion of toads^10,12,13^. Yellow-spotted monitors persist, but remain rare, in areas containing toads^23^.

We tested the aversion of both species of lizard to consuming toads by conducting trials of prey choice at 41 sites across Australia. Those sites were chosen to span the invasion chronosequence for both species of monitors, and extended further west (yellow-spotted monitors) and south (lace monitors) to include uninvaded sites where lizards had not had an opportunity to encounter a toad. In the Austral summer of 2017–2018, we conducted trials along the east coast of Australia to test lace monitors’ responses to toads at 17 sites (5 toad-absent, 12 toad-present; the latter invaded 5–80 years prior to our study). We tested the responses of yellow-spotted monitors at 24 sites across the wet/dry tropics of northern Australia in 2019, including 6 sites where toads were yet to invade, and 18 sites that toads had colonised 6–84 years previously.

We selected 36 lace monitors from toad-absent (n = 14) and toad-present (n = 22) sites for trials. Lizards approached on foot usually ascended a tree. At the tree base we placed a black metal tray (38 × 26.5 cm) containing two food items: a chicken neck (mean ± SE = 27.5 g ± 2.7 g) and a road-killed toad leg (22.7 g ± 1.8 g) randomly orientated on either side of the tray. The observer left the area after deploying a remote sensing camera (Scoutguard SG560K) 1.5 m from the tray and returned after 1 h.

Because yellow-spotted monitors flee readily and rarely climb trees, we quantified their responses from cameras set up in sites known to contain this species. We deployed 384 bait stations (total 16 per site, deployed for 48–72 h over two survey sessions), spaced 100 m apart along transects. Lizards were lured to stations by a non-consumable bait (80 g of sardines in oil) housed in a PVC canister attached to a star picket. A consumable chicken egg was placed at the base of the star picket, and a sardine and toad leg was placed randomly 30 cm to either side under mesh plastic lids that the lizard could access by flipping (to exclude smaller scavenger species). Remote-sensing cameras monitored bait stations, and individual lizards were identified post-trial based on morphological traits to ensure only each lizards’ first interaction with a bait station was included in our analyses. Equipment used on both lizard species was washed and wiped with ethanol after each trial to remove scent cues.

### Analysis

We used Generalised Linear Models (GLM) with a Binomial error distribution and Logit link function to test for changes in the feeding responses of monitor lizards over time. To account for quasi-complete separation of data, we applied Firth Adjusted Maximum Likelihood estimations. Analyses were run separately for each species and prey type (toad leg and non-toxic bait). We used categorical (invasion time: 4 levels; uninvaded, recently invaded, mid-term invaded or long invaded) or binomial predictors (toad status: absence or presence) to test if the feeding responses (binomial: consumed or did not consume) of lizards changed with the presence or duration of sympatry with toads. The levels of invasion used in models were based on the generation times of each lizard species (see^23^ for details). In a further analysis restricted to data from either toad-free or toad-present areas, we asked if lizards were more likely to consume the toad leg or the non-toxic bait. All analyses were conducted in JMP Pro (Ver 14.2).

### Animal ethics

All procedures were approved by the University of Sydney ethics committee (approval 2017/1202) and were carried out in accordance with relevant guidelines and regulations under licence from state and federal wildlife agencies (NSW; SL101977, QLD; *WITK18632517.1*, NT; 63850; WA; 08-002837-2, Commonwealth; RK924).

## Results

**Figure 2:**
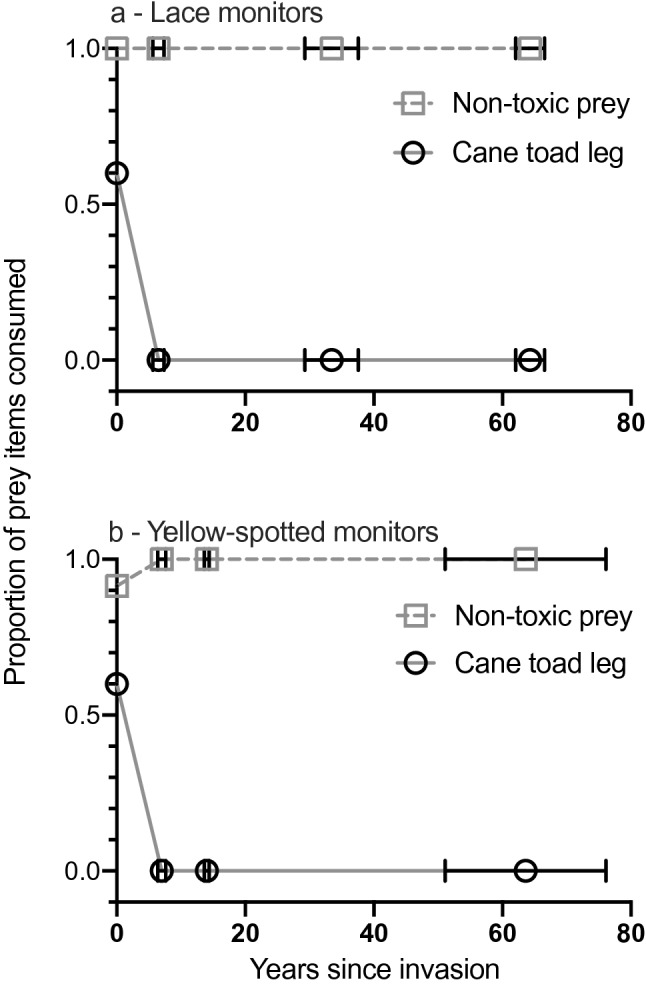
The proportion of cane toad (*Rhinella marina*) legs and non-toxic baits consumed by **(a)** lace monitors (*Varanus varius*) and **(b)** yellow-spotted monitors (*Varanus panoptes*) as a function of the number of years since toads first invaded an area. Lace monitors were offered a chicken neck or toad leg, while yellow-spotted monitors were offered a chicken egg, sardine or toad leg. Error bars represent standard errors surrounding years since cane toads invaded.

Of the 36 lace monitors selected for trials, 8 lizards did not interact with the bait station. Of the remaining 28 trials, all lace monitors investigated both food items (by tongue-flicking) and all consumed the chicken neck. Lace monitors from toad-free sites consumed the toad legs in 60% of trials, but no lace monitors from sites with toads ate a toad leg (Fig. 2). One toad-naïve lace monitor bit the leg multiple times over 40 min but did not consume it. In 43 trials with yellow-spotted monitors, 40 lizards consumed the non-toxic bait choice, but only lizards from areas without toads consumed the toad leg (including 3 lizards that ate the toxic bait, but refused the non-toxic item; Fig. 2). Statistical analysis confirms that the propensity of monitors to consume the non-toxic prey item did not change significantly with the duration of sympatry with toads (lace monitor X^2^_3_ = 0.0, P = 1, yellow-spotted monitor X^2^_3_ = 0.023, P = 0.88) whereas the proportion of monitors that consumed a toad leg was higher in toad-free than toad-present sites (treating toad presence-absence as a dichotomy, lace monitor X^2^_1_ = 14.69, P < 0.0001; yellow-spotted monitor X^2^_1_ = 11.56, P = 0.0007). Even in toad-free sites, however, alternative food types were consumed more often than were toad legs (lace monitor X^2^_1_ = 5.65, P = 0.0175; yellow-spotted monitor X^2^_1_ = 9.97, P = 0.0016). Restricting analysis to sites that contained toads, the duration of toad occupancy of a site was not significantly related to lizards’ responses to toad legs (X^2^_2_ = 0.0, P = 1 for both species).

Although our cameras never recorded a monitor consuming a toad leg in an area containing toads, we observed an adult yellow-spotted monitor pick up and carry away a road-killed toad in March 2017 near Yeppoon, Queensland (an area where toads were common, and had been present for > 50 years) (Fig. 1d). We do not know if the lizard later consumed the toad.

## Discussion

Our results support the hypothesis that taste aversion plays a critical role in buffering vulnerable predators against the arrival of fatally poisonous toads^13,15,24–26^. Vulnerable monitor species can coexist with toads post-invasion because the lizards’ initial willingness to consume toxic toads is abolished soon after toads arrive, and remains minimal for at least 80 years. Interestingly, around 40% of the monitors tested in toad-free areas (and hence, naïve to the dangers posed by toads) refused to consume a toad leg despite readily consuming a non-toxic food item. Clearly, then, monitors treat the novel prey type with caution, a caution that is amplified by continuing contact with toads.

Previous studies on foraging responses of varanid lizards have reported occasional consumption of toads even in areas where toads have been present for many years. For example, a previous trial^14^ reported that one of ten yellow-spotted monitors from an area with toads consumed a toad bait. Similarly, yellow-spotted monitors that developed an aversion to toad-flesh sausages later ate toads^27^, suggesting that the aversion was lost or inconsistent (see^28^). More generally, monitors that handle toads slowly and carefully prior to ingestion are more likely to reject the item^15^. Those observations are consistent with our observation of a monitor carrying a dead toad (Fig. 1d). This behaviour is risky, because even a long-dead toad retains enough active toxin to kill a predator^29^. The lizard that we photographed in Yeppoon may have refrained from actually ingesting the anuran, like one of the toad-naïve lace monitors that we filmed with a camera-trap.

The persistence of taste aversion to toads across the entire invasion history is unsurprising, given the fatal consequences if ingested. However, our spatially extensive sampling is the first empirical evidence of that consistency. In other traits associated with the toad invasion, initial reports of differences between toads from long-colonised areas versus the invasion front in morphology (e.g., leg length^30^ and physiology (e.g., immune responses^31^) proved, on closer examination, to be nonlinear across the course of the invasion. That is, traits in either edge of the toad’s Australian distribution were more similar to each other in these respects than were conspecifics from intermediate parts of the range^32–34^. In the case of behavioural responses of monitor lizards to toads, however, the response appears to be immediate, with no subsequent shifts (Fig. 2).

The strength and consistency of taste aversion revealed by our study may explain why Australian monitors have not evolved physiological resistance to the toads’ toxins. Even a tiny genetic change can confer high resistance, and many lineages of predators from the toads’ native range exhibit that capacity^35,36^. We might thus expect strong selection for these genetic changes in monitor lizards in areas long colonised by toads, but no such changes have evolved^18^. If indeed monitors refuse to eat toads (as suggested by our study), that behaviour removes any advantage to physiological tolerance to the toads’ poisons—and hence, eliminates selection for genetic changes that confer toxin resistance.

Because our data are observational rather than experimental, we cannot identify the proximate mechanisms that underlie the refusal of varanid lizards to consume toads. However, we know that one of the species in this study (*Varanus panoptes*) displays within-population variation in diet^37,38^. Breeding experiments with another vulnerable predator (the quoll *Dasyurus hallucatus*) have revealed a heritable component to such behaviour; the progeny of quolls from toad-infested areas are less likely to consume toad flesh than are the progeny of quolls from toad-free areas^39^. However, taste aversion can also be learned quickly, from even a single episode of nausea^40,41^ and monitors are capable of such aversion learning^15,27^. Hence, the difference in feeding responses between monitors in toad-infested versus toad-free areas may arise from the opportunity (or lack thereof) to learn taste aversion from consuming a small (and hence non-lethal) toad early in life ^42^, rather than (or as well as) by genetic control.

Although both monitor species display long-term intergenerational aversion to toads in areas behind the frontline, the lace monitor has withstood the toad invasion far better than has the yellow-spotted monitor^23^. That difference is not due to initial responses, which were identical in the two species: 60% of toad-naive lizards consumed the bait on their first encounter with this novel prey item. However, other studies have reported that all toad-naïve yellow-spotted monitors ate a second toad that was offered^14^, whereas no lace monitors did so^15^. The more rapid acquisition of aversion may help to explain the greater resilience of lace monitors (compared to yellow-spotted monitors) after toads arrive.

Simple behavioural avoidance may mitigate the impacts of invasive species on other native taxa^43^. Indeed, taste aversion successfully inhibits frogs^44^, fish^45^, skinks^25^ and mammals^24,26,46^ from predating toads. The cues that predict danger may come from invasive predators rather than toxic prey, as in the case of detection and avoidance of invasive mammalian predators by New Zealand lizards^5^. However, behavioural avoidance may not work against all types of invaders, or in all contexts. For example, population-level declines may still occur if even a single age class fails to respond appropriately to the threat posed by an invader^47,48^, or if native taxa fail to recognise invader stimuli^49,50^ or respond inappropriately^51^. Available evidence on monitor lizards suggests that detecting the toxicity of toads—a shift in cognition rather than morphology or physiology—is the key to persistence of these vulnerable predators. As a result, exposing these vulnerable predators to nonlethal doses of toad toxin prior to the arrival of toads, in order to stimulate taste-aversion, can be highly effective in buffering populations of varanid lizards against the otherwise-lethal invaders^13^.

## Data availability

Data available from the Dryad Digital Repository at: 10.5061/dryad.905qftthr^52^.
